# First case of canine paraprostatic infection caused by a high-risk ST147 *Klebsiella pneumoniae* harboring multiple extended-spectrum-β-lactamase genes

**DOI:** 10.1007/s11259-026-11383-1

**Published:** 2026-07-03

**Authors:** Natalia C. Gaeta, Roberto Rodrigues da Rosa Filho, Carine Vieira do Amaral, Willian Seiji Honda, Jéssica Guilhem Alves, Lais Medrado do Nascimento, Carolina de Oliveira Ghirelli, Amanda Haisi, João Pessoa Araújo Jr.

**Affiliations:** 1 Programa de Pós-Graduação em Saúde Única, Universidade Santo Amaro, São Paulo, Brazil; 2 Curso de Medicina Veterinária, Universidade Santo Amaro, São Paulo, Brazil; 3 Clínica Veterinária UNISA, Universidade Santo Amaro, São Paulo, Brazil; 4 Instituto de Biotecnologia. Universidade Estadual Paulista “Júlio de Mesquita Filho”, São Paulo, Brazil

**Keywords:** Genomic analysis, *bla*_CTX−M_, ESBL, Surveillance, One health

## Abstract

Multidrug-resistant (MDR) *Klebsiella pneumoniae*, particularly high-risk clones such as sequence type (ST) 147, have been increasingly reported worldwide. However, data on their occurrence in reproductive disorders of companion animals remain limited. This study describes, for the first time, an extended-spectrum β-lactamase (ESBL)-producing *K. pneumoniae* ST147 isolated from a canine paraprostatic abscess. The isolate (UNISA-DOG-2025) was characterized by antimicrobial susceptibility testing and whole-genome sequencing. Phenotypic analysis revealed an MDR profile, supported by the detection of multiple resistance genes, including β-lactamases (*bla*_CTX−M−8_, *bla*_CTX−M−15_, *bla*_OXA−1_, *bla*_OXA−9_), as well as determinants conferring resistance to aminoglycosides, quinolones, sulfonamides, tetracyclines, and trimethoprim. Several of these genes were located on an in silico predicted conjugative IncM1 plasmid, suggesting potential for horizontal dissemination. Phylogenomic analysis demonstrated close relatedness to globally distributed ST147 strains, predominantly associated with human infections. To our knowledge, this is the first report of *K. pneumoniae* ST147 causing a canine paraprostatic abscess. These findings highlight the clinical relevance of MDR Enterobacterales in canine reproductive disorders and reinforce the need for antimicrobial stewardship and genomic surveillance in veterinary medicine.

**Supplementary Information:**

The online version contains supplementary material available at https://doi.org/10.1007/s11259-026-11383-1.

## Background

*Klebsiella pneumoniae* is an opportunistic pathogen that causes infections in humans and animals, including urinary tract infections, pneumonia, and bloodstream infections. In recent decades, the global emergence of multidrug-resistant (MDR) *K. pneumoniae*, particularly strains producing extended-spectrum β-lactamases (ESBLs) and carbapenemases, has become a major public health concern (Wyres and Holt 2018). The dissemination of antimicrobial resistance in this species is largely driven by mobile genetic elements, which facilitate the accumulation and horizontal transfer of resistance determinants across bacterial populations and ecological niches (Pan and Li 2025).

Among MDR *K. pneumoniae*, several pandemic high-risk lineages have been described, including sequence type (ST) 147, which is widely distributed and frequently harbors ESBL and carbapenemase genes, such as *bla*_CTX−M_, *bla*_NDM_, and *bla*_OXA−48_ (Navon-Venezia et al. 2017). This lineage has been frequently reported in humans worldwide (Aguilar-Ancori et al. 2025) and sporadically in dogs and cats (Ovejero et al. 2017; Lee et al. 2024; Sakauchi et al. 2024; Bassiouny et al. 2026). It is increasingly recognized for its ability to acquire diverse resistance determinants on conjugative plasmids (Navon-Venezia et al. 2017).

In recent years, companion animals have been increasingly recognized as potential reservoirs and sentinels of antimicrobial-resistant Enterobacterales within the One Health interface. ESBL-producing *K. pneumoniae* and other Enterobacterales have been reported in dogs and cats (Menezes et al. 2024), including in Brazil (Sakauchi et al. 2024), suggesting that antimicrobial resistance determinants and high-risk lineages may circulate between human and animal populations. However, the epidemiology, genomic features, and clinical contexts associated with these strains in veterinary medicine remain incompletely understood. Here, we describe a multidrug-resistant ESBL-producing *K. pneumoniae* ST147 isolated from a canine paraprostatic abscess in Brazil. Using whole-genome sequencing and phylogenomic analysis, we highlight the potential role of companion animals in the ecology and dissemination of internationally distributed high-risk clones.

## Case presentation

A 9-year-old male mixed-breed dog was presented to the teaching veterinary clinic at Santo Amaro University, Brazil, in March 2025 with a 2-week history of hematuria, thick, cloudy urine, and pollakiuria. During anamnesis, the owner reported that the patient had been undergoing treatment for cystitis for several weeks. Additionally, in January 2025, the dog had undergone castration and removal of a testicular tumor suspected to be a Sertoli cell tumor. The owner also reported normorexia, normodipsia, and normal defecation.

During physical examination, the dog presented a heart rate of 136 bpm, slightly pale mucous membranes, and mild abdominal pain. An increased subcutaneous volume measuring approximately 4 cm was observed in the right inguinal region, characterized as firm, partially adherent, non-ulcerated, and non-alopecic. Gynecomastia and a pendulous prepuce were also noted. Based on these findings, additional diagnostic tests were requested to further investigate the clinical signs. Enrofloxacin was prescribed (10 mg/kg, SID, for 7 days).

At the recheck examination (eight days later), the owner reported clinical improvement. A follow-up ultrasonographic examination showed, cranial to the prostate, poorly defined hypoechoic areas with heterogeneous echotexture were observed, containing multiple fluid collections with moderate cellularity. The largest measured approximately 8.20 cm in length × 4.84 cm along its major axes. These findings were associated with paraprostatic abscesses or cysts (Supplementary Material 1 available at https://shre.ink/Ljha). Abdominal computed tomography was then requested for surgical planning; however, due to financial constraints, the owner chose to continue treatment at another institution.

One month later (May 23rd), the patient returned due to worsening of the general condition, progressing to marked prostration and anorexia. On physical examination, the animal was poorly responsive to handling and to the environment, with a rectal temperature of 40.2 °C, heart rate of 180 bpm, systolic arterial pressure of 110 mmHg, 5% dehydration, pale mucous membranes, and moderate abdominal pain. Hematological analysis revealed a hematocrit of 37%, total protein concentration of 9.4 g/dL, leukocytosis with a left shift (32,000 cells/mm³, with 1% band neutrophils), and thrombocytopenia (196,000 platelets/mm³). Serological testing for Ehrlichia canis was requested and performed according to the manufacturer’s instructions (Biogal Galed Labs, Israel). An antibody titer of 1:160 was obtained, indicating a moderate positive reaction. Based on these findings, doxycycline was added to the treatment protocol (10 mg/kg, SID, for 30 days), along with ondansetron (0.5 mg/kg, TID, for 5 days), and prednisolone (0.5 mg/kg, SID, for 7 days). The owner was also instructed to resume enrofloxacin (10 mg/kg, SID, for an additional 7 days), together with dipyrone (25 mg/kg, TID, for 5 days), and tramadol (3 mg/kg, TID, for 5 days).

At the recheck examination on June 2nd, the owner reported improvement in the patient’s general condition, with normal appetite, defecation, water intake, and urination. On physical examination, the animal was alert and responsive to handling and to the environment, with a heart rate of 120 bpm, systolic arterial pressure of 170 mmHg, rectal temperature of 38.8 °C, adequate hydration, pink mucous membranes, no lymph node enlargement, and mild abdominal pain. Hematological analysis showed a hematocrit of 30%, total protein concentration of 8.2 g/dL, leukocytosis (39,800 cells/mm³), and a platelet count of 433,000/mm³.

Exploratory laparotomy was performed 14 days after the beginning of doxycycline therapy, following clinical improvement and improvement in hematological parameters. The interval between the first ultrasonographic examination and surgical intervention was related to the owner’s temporary discontinuation of follow-up at our institution and to the need for treatment of ehrlichiosis before surgery.

**Fig. 1:**
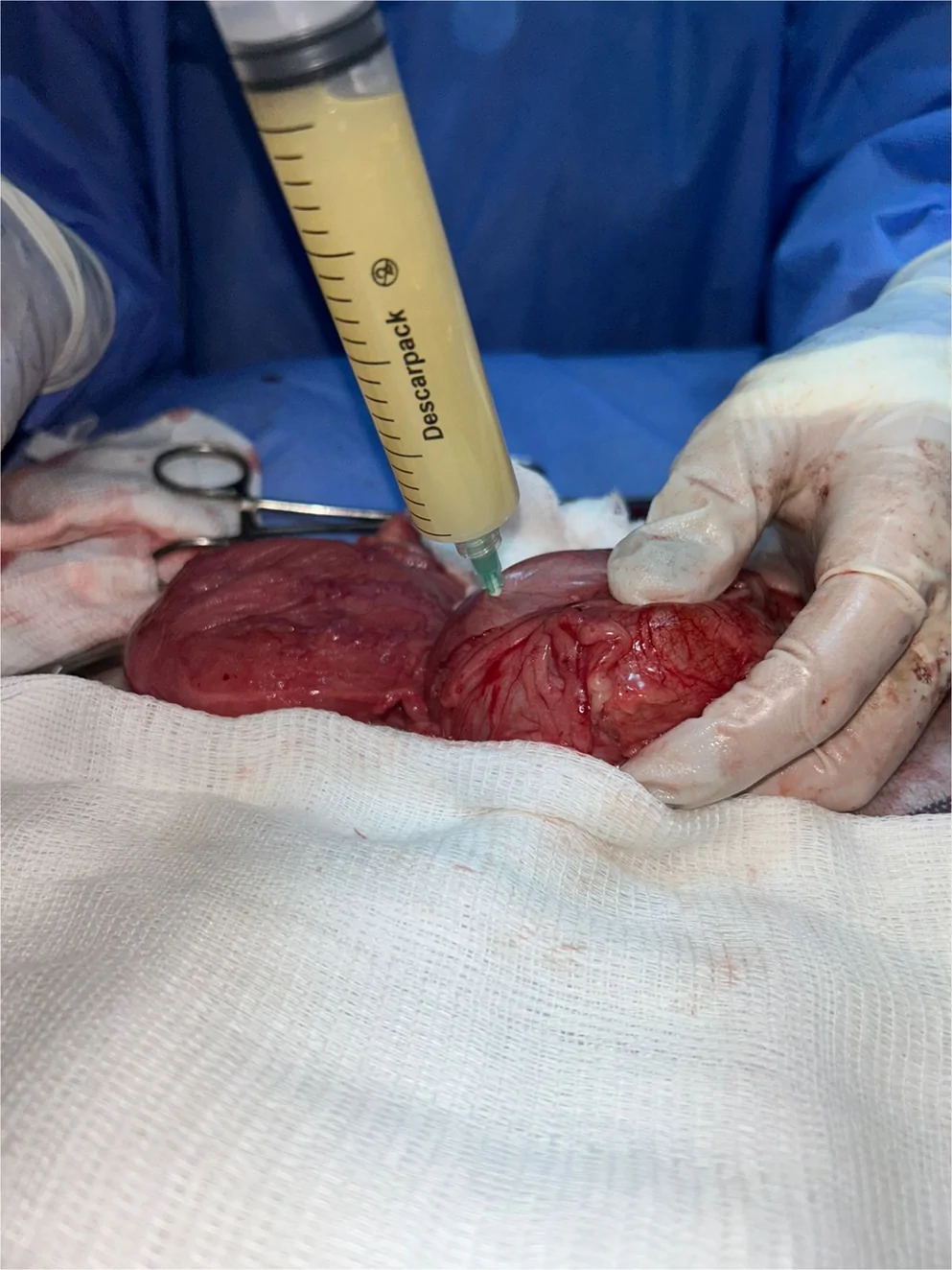
Intraoperative sampling of purulent material from a canine paraprostatic abscess. The collected specimen was submitted for microbiological culture and antimicrobial susceptibility testing

In June 2025, an exploratory laparotomy was performed, during which a purulent content was collected from the confirmed paraprostatic abscess (Fig. 1). The sample was submitted for culture, as well as antimicrobial susceptibility testing. The sample was plated in a 5% sheep blood agar plate, which was incubated at 35 ± 2 °C under aerobic conditions for 24 h. Colonies were separated, and bacterial species were determined using Matrix-Assisted Laser Desorption/Ionization Time-of-Flight (MALDI-TOF; Supplementary Material 2).

Antimicrobial susceptibility was tested using the disk-diffusion test (Kirby-Bauer method) with the following class drugs (DME, Brazil): amoxicillin-clavulanate (AMC; 10 µg), aztreonam (ATM; 30 µg), cefepime (CPM; 30 µg), cefotaxime (CTX; 30 µg), cefoxitin (CFO; 30 µg), ceftazidime (CAZ; 30 µg), ceftriaxone (CRO; 30 µg), ertapenem (ERT; 10 µg), imipenem (IMP; 10 µg), meropenem (MPM; 10 µg), ciprofloxacin (CIP; 05 µg), gentamicin (GEN; 10 µg), nalidixic acid (NAL; 30 µg), sulfonamide-trimethoprim/sulfamethoxazole (SUT; 1.25/23.75 µg), and tetracycline (TET; 30 µg) (CLSI 2025). Furthermore, ESBL production was confirmed by the double-disk synergy test with CPM, CTX, CAZ, CRO, and AMC (CLSI 2025). Multidrug resistance (MDR) was defined as resistance to at least three antimicrobial classes (Magiorakos et al. 2012). Finally, the *Escherichia coli* ATCC 25,922 and the ESBL-producing laboratory strain *Escherichia coli B2C* (GenBank accession #GCA_020644575.1) were used as controls.

The strain was sequenced using DNA extracted with the PureLink^®^ Genomic DNA Mini Kit (Thermo Fisher Scientific, USA) according to the manufacturer’s instructions, and the DNA was stored at −20 °C. DNA concentration was assessed using the Qubit™ dsDNA High Sensitivity Assay Kit (Thermo Fisher Scientific, USA). Library preparation was elaborated with approximately 350 ng of dsDNA with Illumina COVIDSeq (Illumina Inc., San Diego, USA), which was then cleaned with 0.9x Illumina Tune Beads and eluted in 50 µL of resuspension buffer. Sequencing was performed on the MiSeq Platform (Illumina Inc., San Diego, CA) using 2 × 300 bp reads.

Reads were quality assessed, trimmed, and filtered using FastQC v.0.12.1 (https://github.com/s-andrews/FastQC), TrimGalore v.0.6.10 (https://github.com/FelixKrueger/TrimGalore), and Trimmomatic v.0.39 (https://github.com/timflutre/trimmomatic), respectively. De novo assembly was conducted with Unicycler v.0.5.1 (https://github.com/rrwick/Unicycler), and annotation was performed using Prokka v.1.14.0 (https://github.com/tseemann/prokka). The resistome was in silico predicted using CARD (https://github.com/arpcard), Bacmet v.2.0 (http://bacmet.biomedicine.gu.se/), and ResFinder v.4.7.2 (http://genepi.food.dtu.dk/resfinder) databases. PlasmidFinder v.2.1 (https://cge.food.dtu.dk/services/PlasmidFinder/) and mlplasmids v.2.2.3 (https://gitlab.com/sirarredondo/mlplasmids) were used to predict the plasmidome, and MOB-Suite v.3.1.0 (MOB-recon) was used to reconstruct plasmids from draft assemblies (https://github.com/phac-nml/mob-suite#docker-image). Virulome was assessed using the Virulence Finder Database (VFDB v.2.0.1) in the ABRicate tool v.1.0.1. The Sequence Type (ST) was determined using the MLSTFinder online tool (https://genepi.dk/mlstfinder). Finally, Kleborate (https://github.com/klebgenomics/Kleborate) was used to confirm species and assess ICEKp-associated virulence loci, virulence plasmid-associated loci, and capsule (K) and LPS (O) serotype prediction.

The isolated strain (GenBank access #PRJNA1445969) was analyzed using 50 genomes with the same ST, sourced from the BV-BRC database. The genomes were selected and downloaded based on epidemiological data, including country and year of isolation and host source (Supplementary Material 3). In addition, CheckM v.1.2.2 (https://github.com/Ecogenomics/CheckM/) was used to assess sequence quality (≤ 5% contamination, ≥ 90% completeness). Finally, phylogenies were inferred using CSI Phylogeny 1.4 (https://cge.food.dtu.dk/services/CSIPhylogeny/) with default parameters, based on the concatenated alignment of single-nucleotide polymorphisms (SNPs). Phylogenetic analysis was performed with FastTree 2 using the maximum-likelihood algorithm and 1,000 bootstrap replicates. The tree was analyzed using MEGA 11.

Following culture and isolation, pure culture was obtained from the paraprostatic abscess: a hypermucous (Supplementary Material 4a) ESBL-producing *K. pneumoniae* strain, named UNISA-DOG-2025. The phenotypic susceptibility test revealed an MDR phenotype: the UNISA-DOG-2025 was resistant to β-lactams, quinolones, aminoglycosides, sulfonamides, and tetracycline. The strain remained susceptible to CFO, IPM, MPM, and ERT in vitro (Supplementary Material 4b). The quality control strains *E. coli* 25,922 and *E. coli* B2C showed the expected results (sensitive to all drugs tested and ESBL-producing, respectively).

**Fig. 2:**
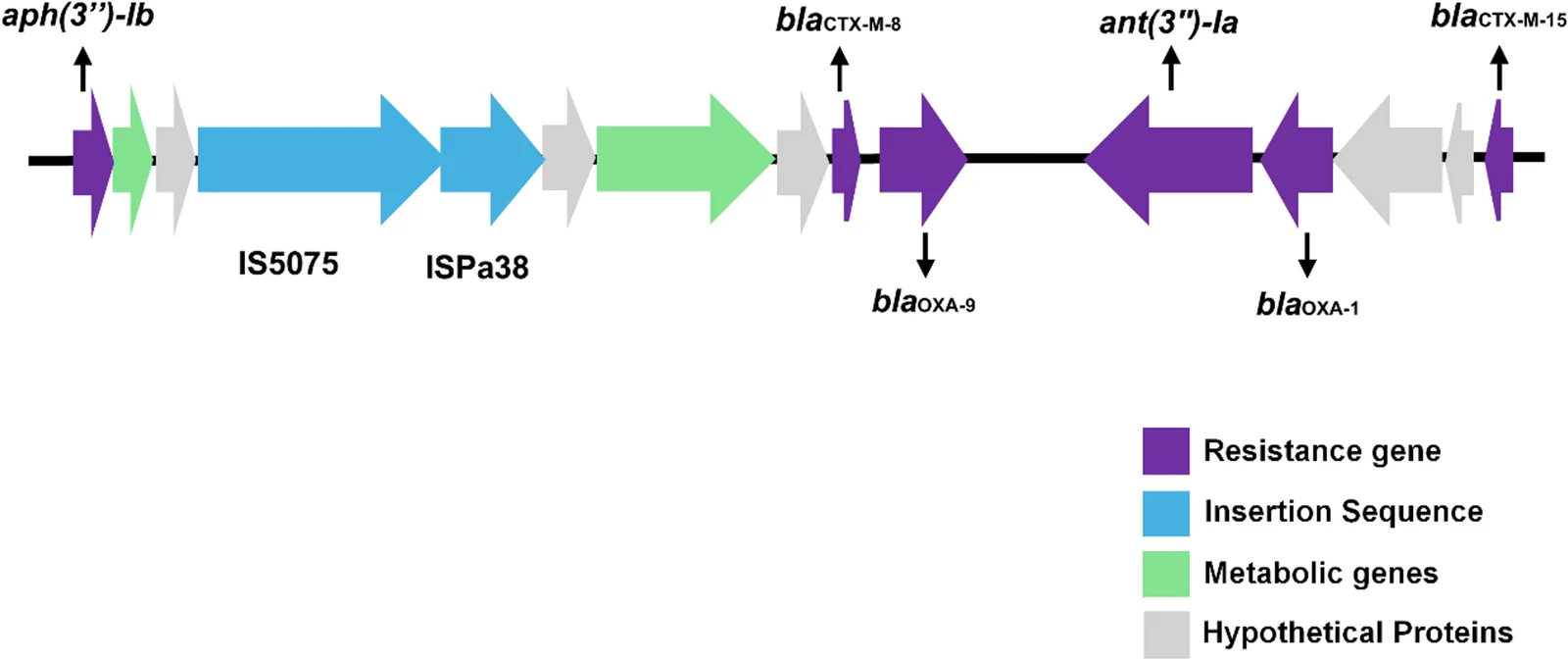
Genetic context and structural organization of resistance genes in *Klebsiella pneumoniae* ST147 (UNISA-DOG-2025), showing the co-localization of *bla*_CTX−M−8_, *bla*_CTX−M−15_, *bla*_OXA−1_, *bla*_OXA−9_, *ant(3″)-Ia* and *aph(3′′)-Ib* flanked by insertion sequences (IS5075 and ISPa38), suggesting mobilization and horizontal gene transfer potential within a multidrug resistance region

The UNISA-DOG-2025 strain was whole-genome sequenced, leading to 2,927,824 reads and approximately 159x coverage (Table 1). The resistome analysis supported the MDR phenotype, identifying resistance determinants for β-lactams (ESBL genes: *bla*_CTX−M−8_, *bla*_CTX−M−15_, *bla*_OXA−1_, *bla*_OXA−9_, *bla*_SHV−11_), aminoglycosides (*aac(3)-IIa*,* ant(3’’)-Ia*,* aph(3’’)-Ib*,* aph(6)-Id*), quinolones (*oqxA*,* oqxB*,* qnrB*,* qnrE1*), sulfonamides (*sul2*), tetracyclines (*tetA*), fosfomycin (*fosA*), and trimethoprim (*dfrA14*) (Table 1). In our strain, *bla*_CTX−M−8_, *bla*_CTX−M−15,_ *bla*_OXA−1_, and *bla*_OXA−9_, in addition to *aac(3)-IIa*,* ant(3’’)-Ia*, and *tet(A)* are harbored in an in silico predicted conjugative IncM1 plasmid. The genetic context of ESBL-related genes is depicted in Fig. 2, and it shows their proximity and upstream flanking by IS5075.

**Table 1:** Genomic data of *Klebsiella pneumoniae* ST147 *(*UNISA-DOG-2025 strain) retrieved from a paraprostatic *abscess* in a dog from Brazil

Characteristics	UNISA-DOG-2025
Source	Paraprostatic abscess
Species	*K. pneumoniae*
ATB resistance profile^a^	AMC, CAZ, ATM, CRO, CTX, CPM, SUT, NAL, CIP, GEN, TET
Genome Size (bp)	5,768,995
No. of CDSs^b^	5,160
G + C content (%)	57.21
N50 (bp)	165,929
tRNAs (*n*)	37
rRNAs (*n*)	5
MLST (ST)^c^	147
WZI	wzi 64
K: O	K64:O2
Resistome	
*β-lactams*	*bla*_CTX−M−8_, *bla*_CTX−M−15_, *bla*_OXA−1_, *bla*_OXA−9_, *bla*_SHV−11_
*Aminoglycosides*	*aac(3)-IIa*,* ant(3’’)-Ia*,* aph(3’’)-Ib*,* aph(6)-Id*
*Fosfomycin*	*fosA*
*Quinolones*	*OqxA*,* OqxB*,* qnrB*,* qnrE1*
*Quinolone point mutations*	*GyrA*:Ser83Ile;*ParC*:Ser80Ile
*Sulfonamides*	*sul2*
*Tetracyclines*	*tet(A)*
*Trimethoprim*	*dfrA14*
*Heavy metals*	*arsABCDR*,* cueR*,* cutA*,* nikBCER*,* pcoABCDERS*,* silABCFPRS*,* znuC*,* zntR*
Plasmidome	IncM1, IncFIB, Col(pHAD28)
Genbank Accession #	PRJNA1445969

Finally, beyond antibiotic determinants, the resistome of UNISA-DOG-2025 encoded tolerance modules to multiple heavy metals (*arsABCDR*, *cueR/cutA*, *nikBCER*, *silABCFPRS*, *znuC/zntR*).

**Fig. 3:**
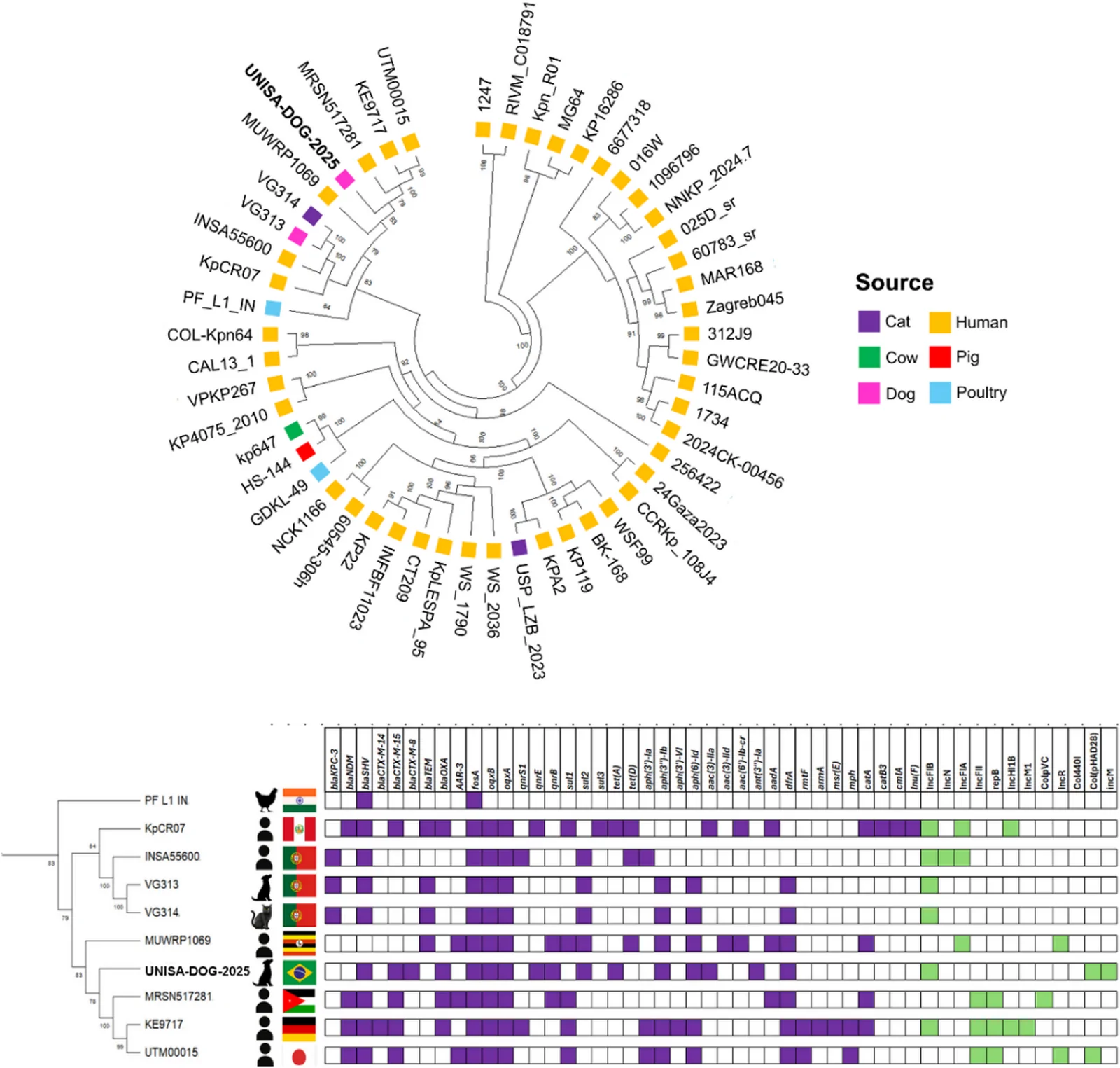
Phylogenomic analysis of *Klebsiella pneumoniae* ST147 (UNISA-DOG-2025) reveals close genetic relatedness to human-derived isolates and a conserved multidrug resistance profile across diverse hosts within a One Health context

Also, the UNISA-DOG-2025 strain belongs to the pandemic high-risk sequence type (ST) 147, and a high-resolution SNP-based phylogenomic analysis revealed that it clustered tightly with a subset of globally distributed ST147 genomes (Supplementary Material 3; Fig. 3a). Interestingly, most isolates were human-derived, and only eight animal-derived ST147 isolates were selected based on the criteria described earlier, of which poultry (India), dog (Portugal), and cat (Portugal) were closest to UNISA-DOG-2025. Given its phylogenetic relatedness to human-associated genomes, UNISA-DOG-25 may have been present in the patient’s household environment or may have colonized the owners, suggesting that these could represent potential sources of infection.

In addition, the resistome analysis revealed a multidrug-resistance profile of most isolates, which clustered with UNISA-DOG-2025 (Fig. 3b). All isolates harbored the *fosA* gene. Moreover, most strains carried resistance genes to quinolones (*oqxA*,* oqxB*), sulfonamides (*sul1*,* sul2*,* sul3*), aminoglycosides (*aph(311)-Ib*,* aph(6)-Id*), and trimethoprim (*dfrA*). Finally, the *bla*_CTM−M−15_ carried by UNISA-DOG-2025 was also detected in four human-derived isolates from Germany, Japan, Peru, and Jordan.

## Discussion and conclusions

The detection of multiple β-lactamase genes highlights the ability of *K. pneumoniae* to accumulate antimicrobial resistance determinants and act as a reservoir of clinically relevant β-lactamases. In veterinary medicine, the emergence of ESBL-producing Enterobacterales has become a growing concern, particularly in companion animals and veterinary hospital environments (Pinto et al. 2025). Among CTX-M enzymes, *bla*_CTX−M−15_ is globally recognized as the most widespread ESBL variant, is frequently associated with high-risk clones of *K. pneumoniae* and has also been reported in companion animals in Brazil (Sakauchi et al. 2024). In contrast, *bla*_CTX−M−8_ has a particular epidemiological relevance in South America (Guzmán-Blanco et al. 2014) and was first described in *P. mirabilis*, *E. cloacae*, *E. aerogenes*, and *C. amalonaticus* from Brazil (Bonnet et al. 2000). This ESBL variant has been reported in bacteria from animals (Abreu et al. 2025) and food sources (Fraccalvieri et al. 2021). In Brazil, studies have detected CTX-M-8-producing Enterobacterales in a variety of animal hosts, including livestock (Nobrega et al. 2021) and companion animals (Sartori et al. 2026), indicating widespread dissemination across ecological niches.

While the detection of *bla*_CTX−M−15_ and *bla*_CTX−M−8_ is relevant due to their distinct global and regional epidemiological importance, their coexistence with other β-lactamase genes (*bla*_OXA−1_, *bla*_OXA−9_, and *bla*_SHV−11_) in the same isolate further increases the clinical and epidemiological significance of this finding. SHV β-lactamases are part of the intrinsic resistome of *K. pneumoniae*, being usually encoded by chromosomal resistance determinants (Bernardini et al. 2019). On the other hand, OXA-type enzymes are often plasmid-mediated and commonly found alongside ESBL genes, contributing to reduced susceptibility to multiple β-lactams. This collection of resistance determinants is commonly observed in high-risk lineages, which highlights the importance of horizontal gene transfer in the evolution of antimicrobial resistance in *K. pneumoniae*. Indeed, the UNISA-DOG-2025 strain belongs to ST147, a high-risk clone globally distributed (Navon-Venezia et al. 2017). It is involved in the spread of *bla*_CTX−M−15_ and other plasmid-mediated ESBLs and carbapenemase-encoding genes (particularly *bla*_NDM_ and *bla*_OXA−48_) in Asia, Europe, and the Middle East (Zautner et al. 2017), as well as in Latin America, including Brazil (Sakauchi et al. 2024). Furthermore, these ESBL and carbapenemase genes in ST147 are commonly carried by incompatibility plasmids, such as IncF, IncR, and IncL/M (Liu et al. 2023). The UNISA-DOG-2025 strain carried a broad-host-range M1 plasmid group, which is usually associated with *bla*_OXA−48_ and *bla*_NDM−1_ genes (Carattoli et al. 2015). In our strain, however, the in silico predicted M1 plasmid harbored the clinically relevant *bla*_CTX−M−8_, *bla*_CTX−M−15,_ *bla*_OXA−1_, and *bla*_OXA−9_ genes.

A limitation of this study is the use of Illumina short-read sequencing alone, which assemblies are frequently fragmented, particularly across repetitive regions and mobile genetic elements. It makes it difficult to confidently link specific resistance determinants to individual plasmids or to reconstruct complete plasmid sequences (Arredondo-Alonso et al. 2017; Juraschek et al. 2021). Therefore, although our approach supports robust identification of resistance genes, in-depth characterization of plasmid and plasmid-mediated dissemination would require complementary long-read or hybrid sequencing approaches (Sierra et al. 2024).

The detection of the *K. pneumoniae* ST147 ESBL-producing strain in a canine paraprostatic abscess highlights the One Health relevance of companion animals as potential reservoirs and sentinels of internationally disseminated high-risk lineages, as also previously reported in Brazilian companion animals (Sakauchi et al. 2024). Moreover, ST147 has been increasingly associated with highly mobile resistance backbones and, in some lineages, resistance-virulence hybrid plasmids, reinforcing its capacity to accumulate and disseminate accessory genomic modules. The co-location of β-lactam, aminoglycoside and tetracycline resistance determinants on the same IncM1 plasmid may facilitate co-selection under diverse antimicrobial exposures, potentially favoring long-term persistence in chronic infection niches and increasing the risk of horizontal transfer.

Metal tolerance loci can act as long-term ecological selectors: metals persist in impacted environments. They may enrich bacteria carrying linked resistance determinants (co-resistance) or shared stress-response/efflux mechanisms (cross-resistance), thereby favoring maintenance and dissemination of multidrug resistance even when antibiotic pressure fluctuates (Baker-Austin et al. 2006). The detection of heavy metal genes in UNISA-DOG-2025 is particularly relevant at the One Health interface, given overlapping selective regions that increase opportunities for horizontal transfer (Wales and Davies 2015).

Finally, the close clustering of the canine isolate with human-derived strains suggests possible shared reservoirs or interspecies transmission routes. Interestingly, the USP-LZB-2023 strain, isolated from a cat with a urinary tract infection in Brazil (Sakauchi et al. 2024), although belonging to the same ST, was positioned in a distinct branch. These findings indicate that, despite sharing the same MLST profile, the canine and feline isolates represent genetically distinct lineages within ST147. The phylogenetic distance observed for the Brazilian feline isolate indicates that multiple ST147 sublineages are circulating in Brazil, likely reflecting independent introduction events rather than direct epidemiological linkage. Also, the results support the hypothesis that high-risk clones, such as ST147, are established in the human population and may be considered emergent in animal populations.

In summary, we describe a multidrug-resistant ESBL-producing *K. pneumoniae* ST147 isolated from a canine paraprostatic abscess, representing, to our knowledge, the first report of this high-risk lineage associated with this clinical condition in dogs. The isolate carried multiple β-lactamase genes, and additional resistance determinants co-located on a broad-host-range, in silico-predicted IncM1 plasmid, highlighting ST147’s capacity to accumulate and disseminate diverse antimicrobial resistance genes. Phylogenomic analysis demonstrated close relatedness with globally distributed ST147 strains, most of which originated from human infections, suggesting potential shared reservoirs and cross-species transmission routes. These findings highlight the importance of companion animals as potential reservoirs and sentinels of internationally disseminated high-risk clones and reinforce the need for integrated genomic surveillance within a One Health framework.

## Supplementary Information

Below is the link to the electronic supplementary material.

Supplementary Material 1 (WMV 4.62 MB)

Supplementary Material 2 (DOCX 14.2 KB)

Supplementary Material 3 (XLSX 13.1 KB)

Supplementary figure 1 (PNG 1.20 MB)

High Resolution Image (TIF 6.02 MB)

## Data Availability

This Whole Genome Shotgun project has been deposited at DDBJ/ENA/GenBank under Bio Project PRJNA1445969.
