# Supplementary material for: First case of canine paraprostatic infection caused by a high-risk ST147 Klebsiella pneumoniae harboring multiple extended-spectrum-β-lactamase genes
Source: Vet Res Commun. 2026 Jul 3;50(5):437. doi: 10.1007/s11259-026-11383-1 (PMC13331917; doi:10.1007/s11259-026-11383-1)
Supplement: Supplementary file 2 — Supplementary Material 2 (DOCX 14.2 KB) [file 11259_2026_11383_MOESM2_ESM.docx]

**MALDI-TOF MS protocol**

After 24 h of bacterial incubation, a single colony was transferred onto a steel target plate using a wooden stick. The spot was then overlaid with 1 µL of 70% formic acid and left to dry at room temperature. Once dry, 1.0 µL of α-cyano-4-hydroxycinnamic acid (HCCA) matrix solution was added, followed by an additional drying period of 5–10 min at room temperature. Instrument calibration was performed using the Bacterial Test Standard protein solution (BTS; Bruker). Each plate included a positive control (*Escherichia coli*) and a negative control consisting of formic acid and matrix only. MALDI-TOF MS analysis was carried out using Flex Control 3.4 software (Bruker Daltonik, Bremen, Germany), in linear mode, with a 337-nm nitrogen laser. Mass spectra were acquired automatically within a range of 2,000-20,000 m/z, and each spectrum was generated from 3,000 laser shots. Plate acquisition followed the manufacturer’s recommendations for microorganism identification using the on-plate protein extraction protocol (Bruker Daltonik, Bremen, Germany). Spectral data were processed with MALDI Biotyper 4.1.70 software (Bruker Daltonik, Bremen, Germany), using the MBT 7311 MPS reference library. Identification scores ≥ 1.7 were accepted as reliable at the genus level, whereas scores ≥ 2.0 were considered reliable for identification at both genus and species levels. All isolates were analyzed in triplicate using the on-plate extraction MALDI-TOF MS protocol.
