# Supplementary figures and images for: First case of canine paraprostatic infection caused by a high-risk ST147 Klebsiella pneumoniae harboring multiple extended-spectrum-β-lactamase genes
Source: Vet Res Commun. 2026 Jul 3;50(5):437. doi: 10.1007/s11259-026-11383-1 (PMC13331917; doi:10.1007/s11259-026-11383-1)

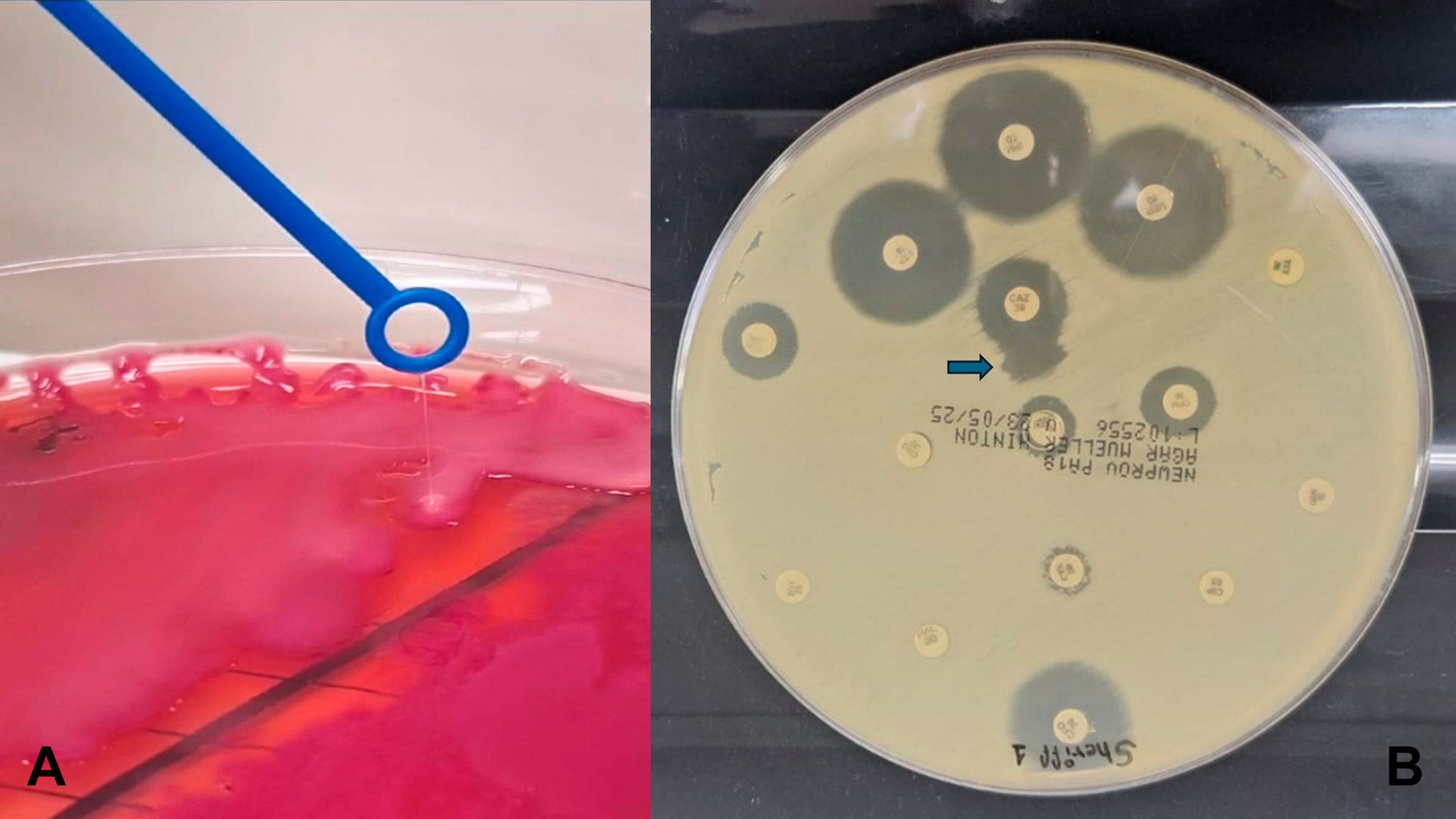

Supplement: Supplementary file 4 — (PNG 1.20 MB) [file 11259_2026_11383_Fig4_ESM.png]

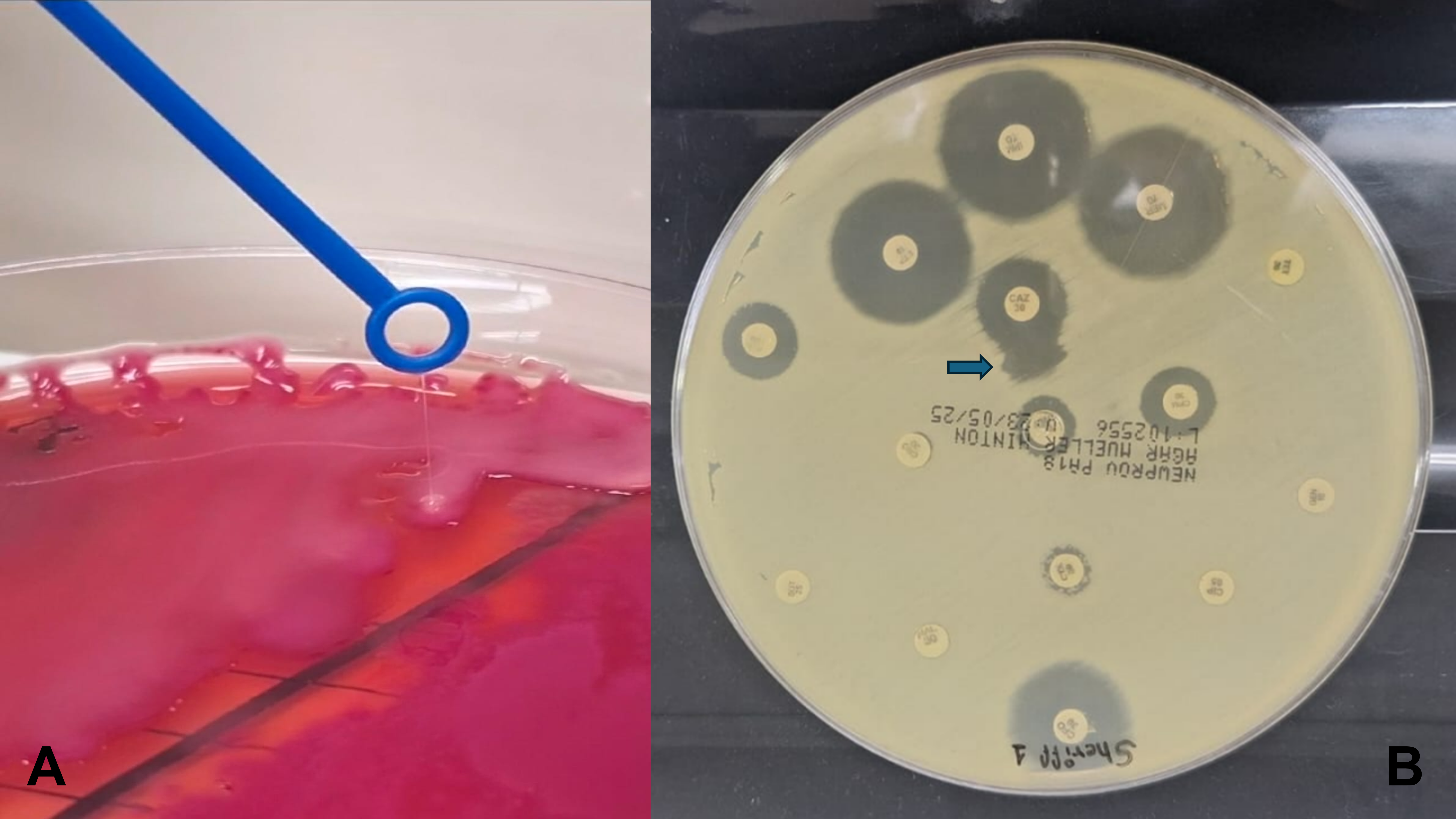

Supplement: Supplementary file 5 — High Resolution Image (TIF 6.02 MB) [file 11259_2026_11383_MOESM4_ESM.tif]
